# Umwelt-Sein. Mutterschaft, Entwicklung und Psychologie, 1930–1990

**DOI:** 10.1007/s00048-020-00277-1

**Published:** 2020-11-05

**Authors:** Susanne Schmidt

**Affiliations:** Institut für Geschichtswissenschaften, Lehrstuhl für Wissenschaftsgeschichte, Humboldt-Universität zu Berlin, Unter den Linden 6, 10099 Berlin, Deutschland

**Keywords:** Umwelt, Mutterschaft, Reproduktionsarbeit, Entwicklung, Psychologie, Antifeminismus, Environment, motherhood, reproductive labor, development, psychology, antifeminism

## Abstract

Dieser Artikel beleuchtet die tragende Rolle, die Umweltdenken und Umgebungswissen für die Legitimation traditioneller Geschlechterrollen im 20. Jahrhundert spielten. Gezeigt wird, auf welche Weise einflussreiche psychologische und psychoanalytische Konzepte der Kindes- und Persönlichkeitsentwicklung Frauen dazu anhielten, sozio-naturale Umwelten herzustellen, ja, selbst Umwelt zu sein. Expertinnen und Experten verschiedener Denkrichtungen und Generationen propagierten ein ganz ähnliches Bild femininer „Environmentalität“, das heißt: der Disposition und Bestimmung der Frau, Umwelten zu erzeugen und zu verkörpern, die eine gesunde Kindesentwicklung ermöglichen und das Wohlbefinden und den Erfolg des Mannes, gar den Erhalt der gesellschaftlichen Ordnung begünstigen sollten. Dieses Konstrukt weiblichen Umwelt-Seins verpflichtete Frauen auf Ehe und Vollzeitmutterschaft und fixierte sie in Raum und Zeit. Sein reaktionärer Gebrauch in Auseinandersetzungen über alternative weibliche Lebensentwürfe demonstriert, dass leitende Konzeptionen von Entwicklung, Wohlergehen und Identität nicht nur androzentrisch, sondern antifeministisch waren.

## Abstract (en)

This article shows how environmental and ambient constructions were used to legitimate traditional gender roles in twentieth-century Europe and the United States. It demonstrates the normative and reactionary character of influential psychological and psychoanalytic theories of childhood and personality development, which instructed women to create, even embody social and emotional environments. This body of thought spanned diverse psychoanalytic schools and extended across generations of psychological experts. They put forth a notion of feminine “environmentality” postulating a woman’s disposition to create, even personify an environment that facilitated normal child development as well as a man’s professional success—and, ultimately, sustained the social order. This construction of women as essentially environmental beings bound them to matrimony and full-time, at-home motherhood, fixing their lives in space and time. It provided a powerful weapon against alternative life-styles and feminist critiques, suggesting that leading conceptions of development, well-being, and identity were not just androcentric, but indeed anti-feminist.

It was becoming clear to me that motherhood was an institution fathered by masculine consciousness. This male consciousness was male unconsciousness. It needed its female partners who were also mothers to stamp on her own desires and attend to his desires, and then to everyone else’s desires. (Deborah Levy, *Things I don’t want to know*)

Männer haben es schwer, darin waren sich die Experten des sechsköpfigen All-Male-Panels einig, das die amerikanische *Vogue *Mitte der 1980er im Kontext antifeministischen Backlashs einberufen hatte (Abb. 1). Wie der New Yorker Rechtswissenschaftler Martin Guggenheim erklärte: „Die Umwelt, in der Männer leben, hat sich [in den vergangenen zwanzig Jahren] verändert. Frauen haben sich so sehr verändert.“ (Rayon 1986: 238).

**Figure 1:**
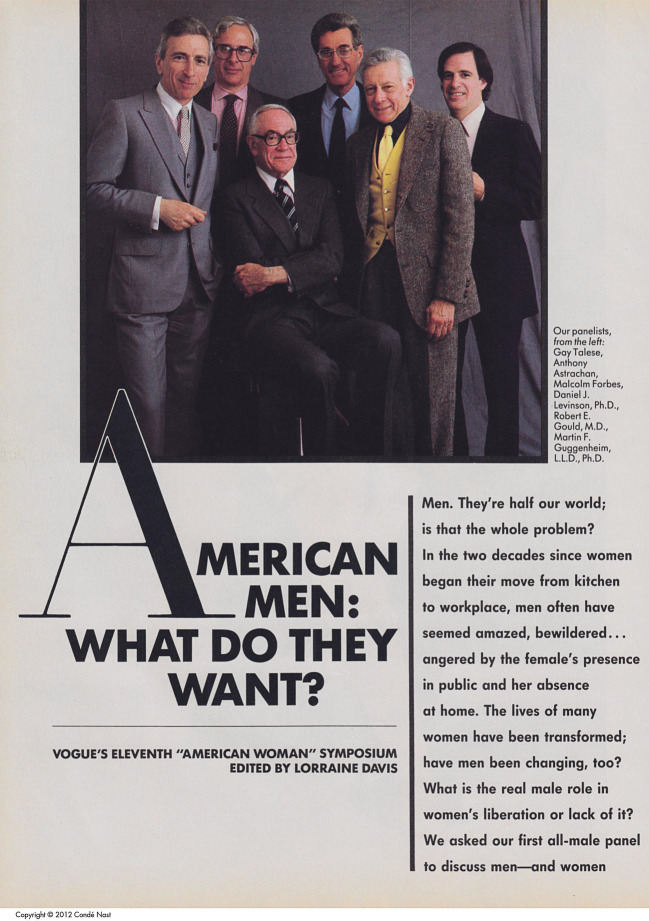

Seine Mitdiskutanten – ein Psychiater, ein Psychologe und drei Journalisten und Schriftsteller – stimmten zu. Aus Sicht des Psychiatrieprofessors Robert Gould handelte es sich um ein unausweichliches Naturgesetz: „Es gibt ein physikalisches Gesetz, demzufolge, wenn ein Teilchen sich ändert, dies sein gesamtes Feld beeinflusst. [Wenn Frauen ihr Leben ändern], müssen auch Männer sich ändern.“ Nicht alle begrüßten das: So begrenzt die Auswirkungen der Frauenbewegung seien, hätten sie doch einen „erheblichen Backlash“ gezeitigt (Rayon 1986: 239). In ihrer Analyse des zeitgenössischen Antifeminismus griffen die *Vogue*-Panelisten auf eine Konzeption der Frau als Medium und Umwelt des Mannes zurück, die Frauen auf ein Leben als Ehefrau und Vollzeit-Mutter verpflichtete. Es waren psychologische und psychoanalytische Forschung und Theorie, die diese weit verbreitete Vorstellung einer feminisierten Umwelt in den USA und in Westeuropa im 20. Jahrhundert maßgeblich propagierten und legitimierten.

Die Her- und Bereitstellung günstiger Umweltbedingungen, die wir hier als „Cocooning“ bezeichnen (Schmidt & Malich 2021), war zentraler Bestandteil analytischer und psychologischer Theorien der Identität und Persönlichkeitsentwicklung. Einige Experten widmeten sich in erster Linie der Bedeutung der Mutter und unterstrichen den Einfluss ihrer Fürsorge auf die Kindesentwicklung. Doch selbst androzentrische Entwicklungsmodelle wie die des Neofreudianers Erik Erikson und seiner Nachfolger, die die weibliche Entwicklung bisweilen kategorisch ausschlossen, richteten sich in erster Linie *an* Frauen, denen sie als Anleitung zu den häuslichen Tätigkeiten der Kindererziehung und Eheführung dienen sollten. Explizit oder implizit wiesen Psychologen und Analytiker Frauen eine Rolle als Gattin und Mutter zu, deren Hauptaufgabe es war, eine optimale, die gesunde Entwicklung des Kindes und das Wohlergehen des Mannes „ermöglichende Umwelt“ (Winnicott) herzustellen und zu repräsentieren.

Nicht nur in der Wissenschaftsgeschichte haben Untersuchungen zu Umwelt und Geschlecht sich bisher auf das weibliche Naturbild der Naturgeschichte und Naturwissenschaften konzentriert (Ortner 1972; Schiebinger 1995; White 2001: 109), während Studien zu Umgebung oder Milieu in den Human- und Sozialwissenschaften sich in erster Linie Prozessen sozialer Regulation und der Anpassung des Individuums an die Gesellschaft widmen (Rose 1999; Roloff 2010). Die Analyse des Umweltbegriffs in Theorien der Persönlichkeitsentwicklung schließt an diese Arbeiten an und führt beide Blickrichtungen zusammen, lenkt das Augenmerk jedoch auf die Hervorbringung und Gestaltung von Umwelten als einer Form der Reproduktionsarbeit oder *care work *(Federici 2017; Cooper 2017; Briggs 2018). Es geht also um Milieu als Appell, um die Aufforderung, die an Frauen erging, Umwelt zu sichern und zu produzieren, ja, selbst Umwelt zu sein.

Beobachtet wird im psychoanalytischen und psychologischen Denken eine Konzeption femininer „Environmentalität“, das heißt: der vermeintlichen Disposition und Bestimmung der Frau, Umwelten zu erzeugen, die eine gesunde Kindesentwicklung ermöglichen und das Wohlbefinden und den Erfolg des Mannes – gar den Erhalt der gesellschaftlichen Ordnung – begünstigen sollten. Im Rückgriff auf ein psycho-physisches Umwelt-Konzept, wie es insbesondere den Begriff der Atmosphäre kennzeichnet (Böhme 2013: 102), bezog Environmentalität sich auf den weiblichen Charakter und den Körper der Frau.^1^ Sie wurde dazu aufgerufen, Umwelten nicht nur zu schaffen oder zu gestalten, sondern durch und durch zu verkörpern: Sie sollte Umwelt *sein*.

Der „Verbindung zwischen den Frauen und der Umwelt“ (Merchant 1980: xix) haben die ökofeministische Bewegung und Analyse besondere Aufmerksamkeit geschenkt. Seit den 1980er Jahren argumentieren Carolyn Merchant, Evelyn Fox Keller und andere Denkerinnen, die ökologische und feministische Perspektiven in Beziehung setzen, für die spezifische Nähe der Frau zur Natur. Sie haben einerseits auf die Verbindung zwischen Umweltzerstörung und der Unterdrückung der Frau hingewiesen und andererseits nahegelegt, dass Frauen auf besondere Weise dafür qualifiziert seien, ökologische Zusammenhänge zu verstehen und gegen die Ausbeutung und Belastung der Natur vorzugehen (Merchant 1980; Keller 1983; Merchant 1995; Mies & Shiva 2014). Auf vielfältige Weise haben historische Studien die Beiträge von Frauen zum Verständnis und Schutz der Natur sichtbar gemacht (zum Beispiel: Gates 1999; Steidl 2019: 259–352) und die spezifischen Zusammenhänge zwischen weiblicher Selbstermächtigung und der Bewahrung der Natur herausgearbeitet, etwa in Untersuchungen zur Rolle von Frauen für Umweltschutzmaßnahmen um 1900 (für einen Überblick: Unger 2012: 75–104; siehe auch Merchant 1984; Norwood 2014).

Im Unterschied dazu fragt dieser Text nach der Verbindung zwischen protektionistischen ökologischen Visionen und traditionellen Geschlechterrollen. Kritikerinnen und Kritiker haben nicht zu Unrecht vor der Verallgemeinerung der Beziehung zwischen Frau und Natur gewarnt, die die Wandelbarkeit und Spezifizität der Relationen zwischen sich verändernden Geschlechterbeziehungen, Ökologie, Wissenschaft und Politik bisweilen verdeckt (Leach & Green 1997; Leach 2007). Die Analyse psychologischer Konzeptionen von Umwelt und der Blick auf einflussreiche konservative, gar reaktionäre Positionen trägt in diesem Sinne zu einem erweiterten Verständnis des Verhältnisses von Umwelt und Geschlecht bei. Die Funktion des Umweltbegriffs als einer geschlechterpolitischen Ordnungskategorie hat in der Forschung bisher wenig Beachtung gefunden. Ihre Problematisierung lädt dazu ein, die „Begeisterung für ökologische Metaphern und Konzepte“ (Güttler 2019: 240) zu hinterfragen, die auch die Wissenschaftsgeschichte und Wissenschaftsforschung seit den 1980er Jahren prägt.

Der Blick auf die Rolle von Frauen in vorherrschenden Theorien der Persönlichkeitsbildung bestätigt und ergänzt Beobachtungen zur Geschlechterpolitik psychologischer Entwicklungstheorien. Carol Gilligan und andere relationale Psychologinnen, feministische Sozialwissenschaftlerinnen, Historikerinnen, Theoretikerinnen und Publizistinnen haben einerseits psychoanalytische Theorien über die Relevanz mütterlicherer Fürsorge als *care*-feministische Analysen *avant la lettre *gelesen und andererseits den Androzentrismus dominanter Modelle der Individuation hervorgehoben, die sich in erster Linie auf Jungen und Männer bezogen, deren Heranwachsen und Selbstfindung sie beschrieben (Gilligan 1982; Chodorow 1985; Herman 1996; Nelson 2015). Ich zeige, dass Konzepte der Mutter-Kind-Beziehung genauso wie Theorien der Ich-Entwicklung Geschlechterunterschiede festschrieben und dass beide gleichermaßen auf normativen Annahmen über *weibliche* Identität und Kapazitäten basierten. Wenn Frauen diese Anweisungen hinterfragten, bestanden Experten darauf – ihrer Ansicht nach drohte eine Veränderung der Rolle der Frau die Entwicklung des Kindes und das Wohlbefinden des Mannes zu beeinträchtigen.

Im Mittelpunkt der folgenden Analyse stehen entwicklungspsychologische Konzepte und Theorien, die sich im Zuge des Booms psychoanalytischer Zugriffe in der Zwischenkriegszeit und seit dem Zweiten Weltkrieg in Westeuropa und Nordamerika verbreiteten. Nicht zuletzt mit der Vertreibung zahlreicher Psychoanalytikerinnen aus Kontinentaleuropa seit den 1930er Jahren gewannen die Ideen Donald Winnicotts, Erik Eriksons, Benjamin Spocks und anderer Entwicklungs- und Sozialpsychologen, Erziehungsratgeber, Theoretiker und öffentlicher Intellektueller insbesondere in Großbritannien und den USA weitreichenden kulturellen und sozialpolitischen Einfluss (Zaretsky 2015; zur Gleichschaltung der Psychoanalyse in Deutschland, vgl. Cocks 1985). Von besonderer Bedeutung sind daher im Folgenden die lebensweltliche, gesellschaftliche Funktion von Entwicklungstheorien, ihre Anwendung in sozial- und bildungspolitischen Kontexten sowie die Positionierungen der Wissenschaftler in öffentlichen Debatten über Arbeit und Familie. Diese bildeten nicht einfach nach- oder nebengeordnete „Popularisierungen“ (Hilgartner 1990), sondern standen in wechselseitiger Abhängigkeit zu wissenschaftlichen Untersuchungen und theoretischen Arbeiten. Psychologinnen und Analytiker reagierten auf gesellschaftliche Veränderungen und formulierten Theorien und Modelle in Auseinandersetzung mit dem Zeitgeschehen. Vielfach nutzten sie ihren Expertenstatus, um sich in öffentliche Debatten einzubringen und abweichende Ansichten zu kritisieren, wobei sie häufig zur Rolle der Frau Stellung nahmen. Maßgebliche Bestände psychologischen Gedankenguts wurden entworfen und gefestigt in der Kritik feministischer Positionen und Praktiken – und auch dies trug wohl dazu bei, dass psychologisches Wissen im öffentlichen Diskurs breiten Anklang fand (Schmidt 2018; Schmidt 2020).

Die integrierte Analyse der öffentlichen und akademischen Sphäre öffnet zugleich den Blick für Gemeinsamkeiten zwischen verschiedenen Schulen und Generationen der Psychoanalyse, die eine disziplinär orientierte Geschichtsschreibung der Psychoanalyse zumeist getrennt betrachtet (Zaretsky 2015). Mit der Fokussierung auf den britischen Analytiker Donald Winnicott, seinen amerikanischen Kollegen Erik Erikson und dessen weniger bekannten Schüler Daniel Levinson – einer der Redner des *Vogue*-Manels – werden einerseits die Verbindungen zwischen den Kontinenten und mit ihnen die Komplementarität zwischen der Objektbeziehungstheorie Londoner Prägung und der Ich-Psychologie der amerikanischen Ostküste sichtbar. Andererseits zeigt sich die Persistenz analytischer Perspektiven insbesondere über die 1960er und 1970er Jahre hinaus, ungeachtet feministischer Kritik und zeitgenössischer Skepsis gegenüber Expertise (Lunbeck 2014; Herzog 2017: 68–72) und jenseits des psychoanalytischen Kanons.

Analysiert werden im Folgenden zunächst Donald Winnicotts Beschreibungen der „normalen“ Mutter als „Umwelt-Mutter“, die dem Konzept weiblicher Environmentalität besonders deutlich Ausdruck verliehen. Definiert als die unausgesetzte Anwesenheit und Verfügbarkeit der Mutter, bezog sich Umwelt-Sein hier nicht nur auf die räumliche, sondern auch auf die zeitliche Ebene. In einem zweiten Schritt soll gezeigt werden, dass Erik Eriksons Phasenmodell der (männlichen) Kindheits- und Persönlichkeitsentwicklung – wiewohl es den weiblichen Lebenslauf zu ignorieren schien – letztlich auf dem gleichen Verständnis einer weiblich codierten, quasi-uterinen Umgebung beruhte. In der Lektüre des Sozial- und Organisationspsychologen Daniel Levinson wird drittens und letztens schließlich deutlich, dass Environmentalität nicht nur eine Anweisung zur Vollzeitmutterschaft war, sondern genauso für Ehe und Partnerschaft galt. Demnach hatte nicht nur die Art und Weise, wie Frauen ihren Alltag organisierten, dem männlichen Wohlbefinden zu dienen, sondern ihre gesamte Lebensplanung sollte auf den Erfolg ihres Partners ausgerichtet sein. Insgesamt demonstriert die Untersuchung, wie tief die Umweltidee im analytischen Denken verankert war, und beleuchtet die konstitutive Funktion des Milieudenkens für die Konzeption und Legitimierung traditioneller Geschlechterrollen. Sie zeigt darüber hinaus, auf welche Weise psychoanalytische und analytisch geschulte Experten sich ausdrücklich gegen feministische Positionen wandten und den Backlash gegen die Frauenbewegung beförderten, legitimierten und theoretisch und wissenschaftlich fundierten.

## Umwelt-Mutter

Der Londoner Kinderarzt und Psychoanalytiker Donald Winnicott (1896–1971) brachte die Rolle, die Frauen im und für den Entwicklungsprozess zugewiesen wurde, besonders deutlich zum Ausdruck: Er beschrieb die Mutter als Teil der „Umwelt“ eines Kindes. Als Vertreter der britischen Objektbeziehungstheorie der 1930er und 1940er Jahre widmete Winnicott sich der Rolle der Mutter-Kind-Beziehung für die Persönlichkeitsentwicklung. In seinen theoretischen Arbeiten ebenso wie in Erziehungsratgebern hob er die Bedeutung der allernächsten, „direkten Umwelt“ für die kindliche Entwicklung hervor, deren Einfluss „von vielen Analytikern [die sich dem Kind als unabhängigen Wesen widmeten] auf subtile Weise unterschätzt“ werde (Winnicott 1974a [1957]: 146).^2^

Winnicott war einer der zentralen Vertreter der Kinder-Psychoanalyse in Großbritannien; sein Werk entstand in enger Auseinandersetzung mit öffentlichen Debatten und mit Blick auf die Alltagsfragen eines breiten Publikums genauso wie auf theoretische Zusammenhänge. Neben Artikeln in Fachzeitschriften und zahlreichen öffentlichen Vorträgen präsentierte der Analytiker in der Zeit von 1943–1962 mehr als 50 Radio-Sendungen in der BBC, von denen die meisten zunächst in Form von Broschüren, später als Teil seines Erziehungsbestsellers *Kind, Familie und Umwelt* (1969) publiziert wurden.^3^ Die neunwöchige Reihe „How’s the Baby?“, erstmals im Herbst 1949 im BBC-Sender „Home Service“ ausgestrahlt, wurde Winnicotts berühmteste Radioserie. Besser bekannt unter dem späteren Titel „The Ordinary Devoted Mother and Her Baby“, wurde sie in teilweise überarbeiteten und erweiterten Versionen in den Jahren 1951, 1952 und 1960 erneut gesendet, unter anderem auch in der *Woman’s Hour *des BBC-Unterhaltungssenders „Light Programme“ (Winnicott 1950; Shapira 2013: 130–135; Karpf 2014: 83, 85). Die Sendungen entstanden in enger Auseinandersetzung mit den beiden BBC-Journalistinnen Janet Quigley und Isa Benzie (Shapira 2013: 120–134; Karpf 2014). Sie wurden zu einer Zeit ausgestrahlt, als die BBC, die während des Zweiten Weltkriegs zu einem bedeutenden kulturellen und gesellschaftlichen Faktor aufstieg, sich maßgeblich an Hausfrauen und Mütter richtete und häufig Familienthemen verhandelte (McKibbin 1998: 471–472; Shapira 2013: 116–119, 121).

In seinen theoretischen ebenso wie publizistischen Arbeiten betonte Winnicott immer wieder: Der Säugling wachse und reife nur, wenn eine so genannte „fördernde Umwelt“ oder „facilitating environment“ existiere – so auch der Titel der klassischen Aufsatzsammlung *Reifungsprozesse und fördernde Umwelt* (Winnicott 1974e [1965]). Diese entscheidende Bedingung einer gesunden Entwicklung sicherzustellen, sei Aufgabe der Mutter, die sich um das Kind kümmere, auf beruhigende Weise anwesend sei, Stabilität, Kontinuität und Verlässlichkeit ermögliche und Unvorhersehbares abwehre. In einem Radio-Beitrag über das „Gedeihen des Kindes“ (1949) verglich Winnicott die Mutter mit einer Gärtnerin. An seine Zuhörerinnen und Leserinnen gerichtet, erklärte er: „Sie geben nur die richtige Menge Erde und Dünger und sorgen dafür, dass die [Blumen-]Zwiebel feucht gehalten wird“ (Winnicott 1969d [1949a]: 24).^4^ Währenddessen sei der Vater – „kaum je zu Hause wenn der Säugling wach ist“ – nicht aktiv an der Pflege und Erziehung der Kinder beteiligt (Winnicott 1969c [1944b]: 95). Seine Aufgabe sei es, die Frau zu „beschütz[en]“ und sie davor zu „bewahr[en]“, sich „nach außen und an ihre Umgebung wenden zu müssen“ (Winnicott 1969d [1949a]: 21; Forrester 2017).

Tatsächlich sollten Frauen die Umweltbedingungen nicht nur herstellen und sichern, sondern selbst „förderliche Umwelt“ sein. Der Analytiker gebrauchte die Begriffe „Umwelt“ und „Mutter“ regelmäßig synonym und unterschied etwa in seiner „Theorie von der Beziehung zwischen Mutter und Kind“ von 1960 nicht zwischen der „Fürsorge der Umwelt“ und der „mütterlichen Fürsorge“ (Winnicott 1974b [1960]: insbes. 50–51, 62–64). An anderer Stelle sprach Winnicott einfach von der „Umwelt-Mutter“ (1974d [1963]: v. a. 96–97).^5^ Umwelt-Sein oder „Environmentalität“ – die Fähigkeit und Verantwortung, günstige Umweltbedingungen zu schaffen, gar zu verkörpern – kam für Winnicott weiblicher Identität gleich, wie er mit seinen einflussreichen Begriffen der „good enough“ oder „ordinary devoted mother“ zum Ausdruck brachte: der Frau, die nichts Außergewöhnliches tat und „nichts weiter als sie selbst“ sei, sondern bloß dem folge, was der Analytiker als ihre innere Natur beschrieb, indem sie sich um ihr Kind kümmerte (1969e [1949b]: 13; 1974a [1957]: 231).

Nicht zuletzt im Anschluss an Winnicotts eigene Darstellung wird die „ausreichend gute“ oder „normale, hingebungsvolle“ Mutter häufig in Abgrenzung zum Ideal der „perfekten“ Mutter verstanden. Im Unterschied zu einer dogmatischen, unerbittlichen und drohenden Tradition der Psychoanalyse, wie sie etwa mit Sigmund Freud, Melanie Klein oder Helene Deutsch in Verbindung gebracht wird, galt und gilt Winnicott als zugewandte und verständnisvolle „Frohnatur“ (Chodorow 1985; Zitat: Nelson 2015: 33).^6^ Im historischen Kontext erschließen sich freilich andere, teilweise entgegengesetzte Bedeutungen des Begriffs der „normalen Mutter“. Während des Zweiten Weltkriegs und in der Nachkriegszeit – jener Zeit, in der Winnicotts BBC-Sendungen produziert wurden – verwies die Betonung von Normalität zunächst auf den Erhalt und die Wiederherstellung der gesellschaftlichen Ordnung. In diesem Zusammenhang wurden Frauen – insbesondere in der Rolle der Hausfrau und Mutter – zum zentralen Symbol der Rückkehr zum „Normalzustand“ (May 2008; Shapira 2013: 135).

Darüber hinaus nutzte Winnicott das Konzept der „gewöhnlichen Mutter“ und den damit einhergehenden Appell an mütterliche Instinkte und Intuition, um sich als Experte zu legitimieren. Dabei ging es nicht nur um die Abgrenzung von „Ammenmärchen“, die eine junge Mutter „an ihren eigenen, wahren Gefühlen zweifeln“ ließen, sondern auch gegenüber anderen akademischen Formen der Expertise (Winnicott 1969a [1943]: 16; 1964b [1943]: 20). Insbesondere richtete sich Winnicott – wie andere zeitgenössische Psychoanalytiker auch – gegen die behavioristischen und hygienischen Erziehungsratgeber der Zwischenkriegszeit, die Anweisungen und Anleitungen ausformuliert und die Mutter-Kind-Bindung hintangestellt hatten. Behavioristische Psychologen, die ein Zuviel an mütterlicher Nähe und Zärtlichkeit kritisierten, betonten die pädagogischen Vorteile öffentlicher Einrichtungen und einer wissenschaftlich fundierten Erziehung gegenüber der Kernfamilie und bestärkten Mütter darin, ihre Kinder tagsüber sich selbst zu überlassen. Auch das Interesse der Hygiene-Bewegung richtete sich weniger auf mütterliche Liebe und Gefühlsfragen als auf die Körper der Kinder und die Ausprägung vorteilhafter Gewohnheiten im Sinne einer umfassenden Sauberkeitserziehung (Urwin & Sharland 1992; Faircloth 2013: 28–31; Shapira 2013: 64–65, 123, 131).

Für das Verständnis von Winnicotts Konzept der Environmentalität von besonderer Relevanz ist schließlich, dass der Begriff der „normalen“ oder „gewöhnlichen“ Mutter die mütterliche Fürsorge als eine Frage des Instinkts darstellte und auf diese Weise nicht nur naturalisierte, sondern auch abwertete. Indem sie Mutterschaft als psycho-biologischen Trieb definierten, delegitimierten Experten seit den 1930er und 1940er Jahren zunehmend Konzeptionen moralischer und politischer Mutterschaft, die die maternalistische Rhetorik des früheren 20. Jahrhunderts geprägt hatten (Plant 2010: insbes. 86–117; Vicedo 2013: insbes. 90–91, 233–234). Im Rahmen von Winnicotts Konzeption der „normalen“ Mutter gab es für Frauen kaum legitime Lebensentwürfe jenseits der Mutterschaft. Der Analytiker pathologisierte ihre Interessen, Tätigkeiten und Verhaltensweisen außerhalb der familiären Sphäre als „abnormal“ und sprach ihnen die Möglichkeit ab, „Ungewöhnliches“ zu tun oder „Außergewöhnliches“ zu erreichen.

Winnicotts Begriff der Normalität war, mit Georges Canguilhem gesprochen, „zugleich ein Zustand und eine Anweisung“ (2004 [1988]: 60). Als normatives und appellatives Modell unterschied sich die „normale Mutter“ deshalb nicht wesentlich vom Idealbild der perfekten „Supermom“. Ihr Gegenpol war vielmehr Superwoman, jene Figur, die britische Feministinnen Anfang des 20. Jahrhunderts im Anschluss an nietzscheanische und individualistische Ideen entworfen hatten, um Genialität, den Drang nach Selbstentfaltung und Willen zur Macht als weibliche Eigenschaften zu reklamieren (Delap 2004).^7^ In seiner Beschreibung der Umwelt-Mutter grenzte Winnicott die reproduktive Tätigkeit klar von der wirtschaftlichen Arbeitsleistung ebenso wie vom kreativen Schaffensprozess ab: Eine Mutter erzeuge oder vollbringe nichts, sie folge lediglich ihren Instinkten. Wie er jungen Müttern erklärte, war Kindererziehung weniger produktiv als selbst die trivialsten Hobbies: „Einige von Ihnen haben sich künstlerisch betätigt. Sie haben gezeichnet oder gemalt, Tonplastiken modelliert oder Pullover gestrickt und Kleider genäht. Wenn Sie dies alles taten, waren die entstandenen Dinge wirklich von Ihnen gemacht. Bei den Säuglingen ist das anders. Sie wachsen von selbst, und Sie haben nur für eine günstige Umwelt zu sorgen“ (Winnicott 1969d [1949a]: 24–25).

Wie der Begriff des Gewöhnlichen („ordinary“) die Negation von Individualität implizierte, so entsprach auch Environmentalität nicht einfach weiblicher Identität, sondern negierte vielmehr das Potenzial für weibliche Selbstentfaltung und die Entwicklung einer eigenständigen Persönlichkeit. Winnicott zufolge gab eine Frau im Moment der Empfängnis ihre Identität auf: Sie „verschiebt einen Teil des Gefühls für ihr Selbst auf das Kind“ und passt sich „an die Bedürfnisse des Säuglings“ an (1974b [1960]: 68, 69; 1969g [1949d]: 32; Ogden 2007: 77). Als Umwelt des Kindes hatte die Mutter keine unabhängige Persönlichkeit, sondern war stattdessen als Funktion und Bezugsperson des Kindes definiert, als „Objekt“, nicht Subjekt im psychoanalytischen Sinne (Dever 1998: 67).

## Die Zeit anhalten

Ein zentrales Charakteristikum der Umwelt-Mutter war denn auch ihre Unsichtbarkeit. „Befriedigende mütterliche Fürsorge wird nicht bemerkt“, hieß es in Winnicotts Ausführungen über die Mutter-Kind-Beziehung (1974b [1960]: 67). Die Verborgenheit der mütterlichen Fürsorge war gar Ausweis der erfolgreichen Anpassung der Mutter an ihr Kind, wie der Analytiker an anderer Stelle erklärte: „Wir wissen […], dass der Säugling die Umwelt nicht als Umwelt wahrnimmt, besonders dann nicht, wenn die Umwelt gut oder gut genug ist. […] [D]as, was wir eine gute Umwelt nennen, wird als selbstverständlich hingenommen“ (Winnicott 1974a [1957]: 146; vgl. Winnicott 2017b [1955]: 62).

Ein solcher Lobpreis der geräuschlos funktionierenden Frau brachte Kritik am Ideal der Vollzeitmutterschaft zum Schweigen und pathologisierte Zweifel und Probleme als Ausweis „weiblicher ‚Hysterie‘ und Überreaktion und schädlicher *Präsenz*“ (Menkedick 2020: 49, Hervorhebung im Original). Für eine gute, „normale“ Mutter, so legte Winnicott nahe, sollte es überhaupt keine Komplikationen geben. Die Auffälligkeit der Umwelt sei ein Zeichen mütterlichen Versagens („environmental failure“) und ein Störfaktor für die kindliche Entwicklung. Wiederholt thematisierte Winnicott den destruktiven Charakter, den eine „Umwelt“ habe, sobald sie vom Kind wahrgenommen würde: „Die Umwelt löst tatsächlich Reaktionen aus, wenn sie in irgendeiner wichtigen Hinsicht versagt“ (Winnicott 1974a [1957]: 146; siehe auch 1960 [1956]: 397–398; 1974c [1962]: 85–87). Als Winnicott seine Überlegungen zu den Konflikten zwischen Säugling und „Umwelt“ im Rahmen eines BBC-Vortrags ausführte, veranlasste die Produzentin Isa Benzie ihn zu umfassenden Änderungen. Benzie erklärte: „Wenn ich eine Mutter wäre […], würde mir dieses Skript den deutlichen Eindruck vermitteln, dass ich – so stelle ich es mir vor – die Verantwortung für die Probleme des Babys trage. […] Was mir Sorgen bereitet, ist mein Verdacht, dass Sie denken, dass sie (also Mütter) irgendwie schuld sind“ (zitiert nach Shapira 2013: 133; vgl. Winnicott 1969h [1950]).

Winnicott war gegen „wages for housework“ (Federici 2017). In einem Nachwort zu den BBC-Beiträgen bestand er darauf, dass der gesellschaftliche Beitrag der Mutter nicht beziffert oder auch nur gewürdigt werden könne, „weil er so ungeheuer groß ist“ (Winnicott 1969j [1957]: 231). Dies galt für die Mutter-Kind-Beziehung genauso wie auf ökonomischer Ebene. Verlief die Entwicklung eines Kindes „normal“, so behielt es sich seine Unkenntnis, gar Geringschätzung des Einsatzes der Mutter sein Leben lang bei. Wie der Analytiker betonte, schuldete das Kind der Mutter „weder Dank noch Lob“; ihre Fürsorge war spontan und daher selbstverständlich (Winnicott 1969j [1957]: 231). Dieser Aspekt wurde besonders dann relevant, wenn es um das schlussendliche Herauswachsen aus der symbiotischen Beziehung ging. Denn zumindest für einen Jungen war die Mutter nicht Ziel der Entwicklung, sondern lediglich ihr Mittel: „[D]er Mann […] kann offensichtlich nicht dadurch in Übereinstimmung mit seiner Mutter kommen, dass er selbst wieder zur rechten Zeit Mutter wird“ (Winnicott 1969j [1957]: 232). Für Jungen und Männer war die Mutter, in entwicklungspsychologischer Begrifflichkeit, ein „Durchgangsobjekt“ der Persönlichkeitsbildung, von dem es sich früher oder später abzulösen galt.^8^

Wurde, wie spätere Analytikerinnen beobachteten, die männliche Entwicklung „anhand der Entfernung von der Mutter gemessen“ (Gilligan 1982: 184; Chodorow 1985), so war die weibliche Identität demgegenüber unveränderlich. Für Frauen bedeutete Environmentalität, dass ihre Existenz als festgelegt und gleichbleibend definiert war, entsprechend der Idee des stabilen Ökosystems, die, in der Naturphilosophie der frühen Neuzeit ausformuliert, eine der Grundannahmen noch des späten modernen lebenswissenschaftlichen und milieutheoretischen Diskurses konstituierte (Egerton 1973; Canguilhem 2013 [1966]). Die Konzeption der Mutter als Milieu setzte auf grundlegende Weise ihre Stabilität und Konstanz voraus, die sich am deutlichsten in ununterbrochener Anwesenheit ausdrückten.

Mutterschaft, definiert qua Präsenz, war ein unausgesetzter, permanenter Zustand. Körperliche Nähe – weder Charaktereigenschaft noch Tätigkeit, sondern Anwesenheit an sich – war das zentrale Merkmal von Environmentalität. In *New Era in Home and School*, dem Organ der internationalen reformpädagogischen Organisation New Education Fellowship, forderte Winnicott:

Es muss ungehinderten Zugang zum lebendigen Körper der Frau geben. Ohne die lebendige Gegenwart der Mutter sind ihre besten Kenntnisse nutzlos. […] Ihre Lebendigkeit und körperliche Handhabe bieten ein lebenswichtiges psychologisches und emotionales Milieu, entscheidend für das frühe emotionale Wachstum des Kindes. (1964a [1947], 89–90)^9^

Der Kinderarzt war ein Verfechter des Stillens, jener essenziellen Praxis des Selbstverständnisses moderner „intensiver Mutterschaft“ (Hays 1995; Faircloth 2013; Schiebinger 1995: 101–111). Zu einer Zeit, als Flaschennahrung sich im Zuge der Medikalisierung von Schwangerschaft und Geburt in Großbritannien – wie auch in den USA – zunehmend verbreitete (Apple 1987; Wolf 2001; Faircloth 2013: 39–40), widmete Winnicott dem Stillen zahlreiche seiner Rundfunkbeiträge und erklärte es zur grundlegenden Metapher für die Mutter-Kind-Beziehung: „Ich meine, dass es lehrreich sein müsste, sich vorzustellen, dass alles, was Sie aus Liebe zu ihrem Kind tun, ebenso von ihm aufgenommen wird wie die Nahrung“ (1969f [1949c]: 42).

Winnicotts Beschreibung der Umwelt-Mutter war repräsentativ für die psychoanalytische Abgrenzung von Mutter und Außenwelt (Dever 1998: 65) und schloss an breitere Topoi femininer Innerlichkeit und Häuslichkeit und der Trennung zwischen Privatem und Öffentlichem an. Der Analytiker beschrieb die mütterliche Perspektive als nach innen gerichtet und „eingeengt“ in ihren Interessen. Wenn die schwangere Frau „der Überzeugung [war], dass der Mittelpunkt der Welt sich in ihrem eigenen Körper befindet“ (Winnicott 1969a [1943]: 15), so kümmerte die Mutter „sich um das Innere eines Kreises, den sie mit ihren Armen bilden kann und in dessen Zentrum das Kind ist“ (Winnicott 1969d [1949a]: 21; vgl. ders. 1974b [1960]: 68–70 sowie 1960 [1956]).

Winnicott verordnete (werdenden) Müttern „reproduktive Entsagung“ (Ettorre 2000: 407–409). Sie sollten ihre beruflichen und politischen Interessen ebenso zurückstellen wie sportliche und gesellige Aktivitäten: „[Bevor eine Frau ein Kind erwartet], kann sie ein Mensch mit vielen Interessen gewesen sein, vielleicht im Geschäftsleben gestanden haben, sich mit Politik befasst oder leidenschaftlich Tennis gespielt haben, oder sie kann ständig zu Tanzereien oder Partys gegangen sein. […] Aber früher oder später wird sie […] schwanger“ (Winnicott 1969a [1943]: 15). Nun ignorierte sie die Außenwelt zu einem Grad, der, wie der Psychologe nahelegte, bei einem Mann Anzeichen einer Pathologie wäre – „einer Bewusstseinstrübung oder sogar einer Störung auf tieferer Stufe, wie etwa einer schizoiden Episode“ (Winnicott 1960 [1956]: 395–396). Auf welche Weise seine Ratschläge das Wohlergehen der Mutter tatsächlich beeinträchtigten, interessierte Winnicott offenbar wenig.^10^

Die Gegenwart der Mutter fasste der Analytiker im Konzept des „Haltens“ des Kindes zusammen, einem psycho-physischen Akt, wie er aus psychiatrischer Sicht als typisch für die Erzeugung von Atmosphäre galt (Tellenbach 1968) und dem Winnicott eine zentrale und symbolische Funktion zuschrieb.^11^ Es bezeichnete nicht nur „das wirkliche, physische Halten des Säuglings“, sondern die gesamte Tätigkeit der Mutter – oder, in Winnicotts Worten, „die gesamten Umwelt-Vorkehrungen“, auf räumlicher genauso wie zeitlicher Ebene (1974b [1960]: 56, siehe auch ebd.: 56–58, 62–70).^12^ Tatsächlich hat der Akt des Haltens neben der physischen immer auch eine chronologische Dimension, muss man doch jemanden (oder etwas) für eine bestimmte Zeit ergreifen, damit es sich tatsächlich um „Halten“ handelt. Für Winnicott war dieser temporale Aspekt zentral und er bezifferte die Dauer des Haltens genau, als Dauerhaftigkeit: mit 24 Stunden (Forrester 2017).

Die Mutter schirmte das Kind vor den Einflüssen der Außenwelt und Außenzeit ab: vor der Zeit der Uhren und Kalender, vor geregelten Still- und Fütterzeiten – Winnicott war ein Verfechter des Stillens nach Bedarf – genauso wie vor den Arbeitszeiten, Wochenenden und Feiertagen, ja, selbst vor dem Unterschied zwischen Tag und Nacht. Als „haltende Umwelt“ (Winnicott 1974b [1960]: 55–64, 70) absorbierte die Mutter den Einfluss der Zeit auf Kosten ihres eigenen mentalen und körperlichen Wohlbefindens, indem sie auf Schlaf, Erholung und Auszeiten ebenso verzichtete wie auf Unterhaltung und Geselligkeit (Winnicott 1960 [1956]; Ogden 2007: 78). Ihre unablässige Präsenz und der Halt, den sie dem Kind in körperlicher und emotionaler Hinsicht bot, waren gewissermaßen eine Fortsetzung der Schwangerschaft mit anderen Mitteln. Seinen Hörerinnen und Leserinnen erläuterte Winnicott: „Sie haben das Kind empfangen, und von dem Augenblick an wurde es ein Bewohner Ihres Leibes. Nach der Geburt wurde das Kind ein Bewohner innerhalb Ihrer Arme“ (Winnicott 1969d [1949a]: 21).

Winnicotts Beschreibungen femininer Fürsorge und Verfügbarkeit stilisierten und zementierten die Rolle der Frau als Hausfrau und Mutter. In dem BBC-Beitrag „Their Standards and Yours“ („Kinder haben andere Maßstäbe“), der im Mai 1944 gesendet wurde, tat der Analytiker die Möglichkeit, dass Frauen ein selbstbestimmtes Leben außerhalb von Ehe, Familie und Haushalt führen könnten, mit der Behauptung ab, die wahre Unabhängigkeit der Frau sei allein im Haus zu finden: „Das Gerede von den Frauen, die [von den Truppen zurückkehren und] nicht [mehr] Hausfrauen [sein wollen], erscheint mir barer Unsinn, denn nirgends als in ihrem eigenen Hause hat die Frau so viel zu sagen. Nur zu Hause ist sie frei“ (Winnicott 1969b [1944a]: 102; Radiomanuskript zitiert nach Shapira 2013: 126). Gut zwanzig Jahre später, bei Übernahme des Textes in den Ratgeber *Kind, Familie und Umwelt* wurde die Bezugnahme auf den Weltkrieg gestrichen. Diese Aktualisierung des Textes transformierte Winnicotts Idealisierung und Rechtfertigung der Hausfrauenrolle in eine Kritik an den Ideen und Forderungen der Frauenbewegung, die im Laufe der 1960er Jahre zunehmend an gesellschaftlichem Einfluss gewann. In seinem Vortrag „Apropos Feminismus …“, 1964 auf einem Treffen der Progressive League – einem Freidenker-Verband, dem Winnicott nahestand – zum Thema „The Sexes Today“ gehalten (Winnicott 2009a [1964]), betonte der Kinderarzt die Unterschiede zwischen den Geschlechtern und sprach von der Frauenbewegung als einer „mehr oder weniger krankhaften Erscheinung“ (1986 [1964]: 188).^13^ Aus dem psychoanalytischen Entwicklungsmodell leitete er ab, dass eine feministische Einstellung Ausdruck von Missgunst und „Neid“ auf die Entwicklungsmöglichkeiten des Mannes sei, „denn [anders als Frauen] werden Männer im Laufe ihres Lebens immer mehr sie selbst, immer einzigartiger“ (1986 [1964]: 192–193).

Die von Winnicott porträtierte „Umwelt-Mutter“ war kennzeichnend für zeitgenössische und spätere Konzeptionen der Entwicklung und Persönlichkeitsbildung. Dabei trat sie in vielen Fällen nur am Rande in Erscheinung; außerhalb der Bindungspsychologie war der Begriff der „mütterlichen Umwelt“, selbst wo er verwendet wurde (hier: Erikson 1963: 66), kaum leitend. Doch gerade androzentrische Entwicklungstheorien, die sich – wie Winnicott kritisiert hatte – in erster Linie dem individuellen Entwicklungsgang widmeten und der Rolle Mutter wenig Aufmerksamkeit schenkten, basierten auf der Annahme femininer Environmentalität. Sie erklärten die Mutter – ebenso wie die Ehefrau – zur Bedingung normalen kindlichen Wachstums und des männlichen Wohlbefindens.

## Die zweigeteilte Entwicklung

Als unsichtbare Andere (de Beauvoir 1990 [1949]) bildeten Frauen das konstitutive Außen eines immanent maskulinen Begriffs der Individuation und Selbstentfaltung – auch und gerade wenn ihre Rolle ausdrücklich nicht thematisiert wurde. Die von dem Psychoanalytiker und öffentlichen Intellektuellen Erik Erikson (1902–1994) vorgelegte Stufentheorie der psychosozialen Entwicklung aus *Kindheit und Gesellschaft* war eines der einflussreichsten Modelle der Identitätsentwicklung in den USA der Nachkriegszeit und ist bis heute klassischer Lehrbuchstoff der Psychologie, Pädagogik und verwandter Bereiche (Tab. 1). Hervorgegangen aus Eriksons Auseinandersetzung mit Fragen der Ich-Psychologie und Ich-Entwicklung trug *Kindheit und Gesellschaft *– im Original zuerst 1950 veröffentlicht – maßgeblich zu seinem Ruf als „Schöpfer der Identität“ (Friedman 1999) bei.

**Table 1:** 

VIII	Reife	–	–	–	–	–	–	–	Ich-Integrität gegen Verzweiflung
VII	Erwachsenen-Alter	–	–	–	–	–	–	Zeugende Fähigkeit gegen Stagnation	–
VI	Frühes Erwachsenen-Alter	–	–	–	–	–	Intimität gegen Isolierung	–	–
V	Pubertät und Adoleszenz	–	–	–	–	Identität gegen Rollenkonfusion	–	–	–
IV	Latenz	–	–	–	Leistung gegen Minderwertigkeitsgefühl	–	–	–	–
III	Lokomotorisch-genital	–	–	Initiative gegen Schuldgefühl	–	–	–	–	–
II	Muskulär-anal	–	Autonomie gegen Scham und Zweifel	–	–	–	–	–	–
I	Oral-sensorisch	Urvertrauen gegen Misstrauen	–	–	–	–	–	–	–
–		1	2	3	4	5	6	7	8

Eriksons Stufenmodell der Entwicklung schloss an das Konzept einer adoleszenten „Identitätskrise“ an, das er Mitte der 1940er in der Arbeit mit amerikanischen Kriegsveteranen entworfen hatte. Ungeachtet seiner späteren, enthusiastischen Rezeption in der *counterculture* der 1960er Jahre betonte Erikson – nicht anders als Winnicott – die Bedeutung traditioneller Erwerbsstrukturen und Geschlechterrollen in der Kriegs- und Nachkriegszeit (Friedman 1999: 160–161). Doch während er mit seinem britischen Zeitgenossen ein Interesse an Fragen der Entwicklung teilte, stellte Erikson Fragen der individuellen Entwicklung in den Vordergrund. Mit einem Wink Richtung Winnicott bezeichnete er den Einfluss der Mutter-Kind-Beziehung als „Gemeinplatz“ (Erikson 1963 [1950]: 208; vgl. Winnicott 1953).

Im Anschluss an Freuds Theorie der psychosexuellen Entwicklung – von der oralen über die anale zur phallischen, latenten und genitalen Phase – fokussierten Eriksons „Eight Stages of Man“ oder „Acht Phasen des Menschen“ auf die frühe Kindheit, verfolgten die Persönlichkeitsentwicklung aber letztlich über die Jugendzeit bis ins junge und – in groben Zügen – späte Erwachsenenalter (Erikson 1965 [1950]: 241–270). Der Entwicklung von Vertrauen, Autonomie und Leistungsdenken in den ersten Lebensjahren folgten eine adoleszente „Identitätskrise“ (Stufe 5) und die Herausbildung der Fähigkeit zu erwachsener Intimität und Generativität (Stufen 6 und 7). Auf der achten und letzten Stufe der Reife bezeichnete Erikson mit dem Stichwort der „Integrität“ nicht nur die Akzeptanz des eigenen Lebensentwurfs, sondern auch die Identifikation mit den Werten der eigenen Gesellschaft: Für den Analytiker bedeutete Entwicklung den Erhalt der bestehenden sozialen Ordnung.

Eriksons Hauptaugenmerk galt dem Wachstum von Jungen und Männern. Wie der Psychiater und Analytiker Ives Hendrick bereits 1950 beobachtete, handelte es sich bei den „Acht Stufen“ um einen Entwurf des männlichen, nicht des weiblichen Lebenslaufs (Senn 1950: 46–47). Das für sein Entwicklungskonzept so zentrale Konzept der Identitätskrise hatte Erikson in der Behandlung traumatisierter Soldaten entwickelt (Friedman 1999: 160–161). Doch als Modell der Kindheit in der Nachkriegszeit betrafen die „Acht Stufen“ Fragen der Erziehung ebenso wie der Entwicklung. Eriksons Biograph Lawrence Friedman hat darauf hingewiesen, dass die „Acht Stufen“ die Beziehung zwischen Eltern und Kindern verhandelten. In der Tat sah Erikson selbst in der intergenerationellen Dimension den Unterschied zwischen seinem Modell und Freuds Theorie der frühen Kindheit (Friedman 1999: 222, 225; Erikson 1961: 151). Im erzieherischen und bildungspolitischen Gebrauch wurde deutlich, dass Eriksons populäres, androzentrisches Entwicklungsmodell auf einem Konzept traditioneller Weiblichkeit beruhte, das Winnicotts Beschreibung weiblicher Environmentalität entsprach. Psychoanalytikerinnen genauso wie Praktikerinnen und Entscheidungsträger imaginierten mütterliche Präsenz als Bedingung gesunder männlichen Entwicklung, ja als Grundlage einer intakten Gesellschaft.

**Figure 2:**
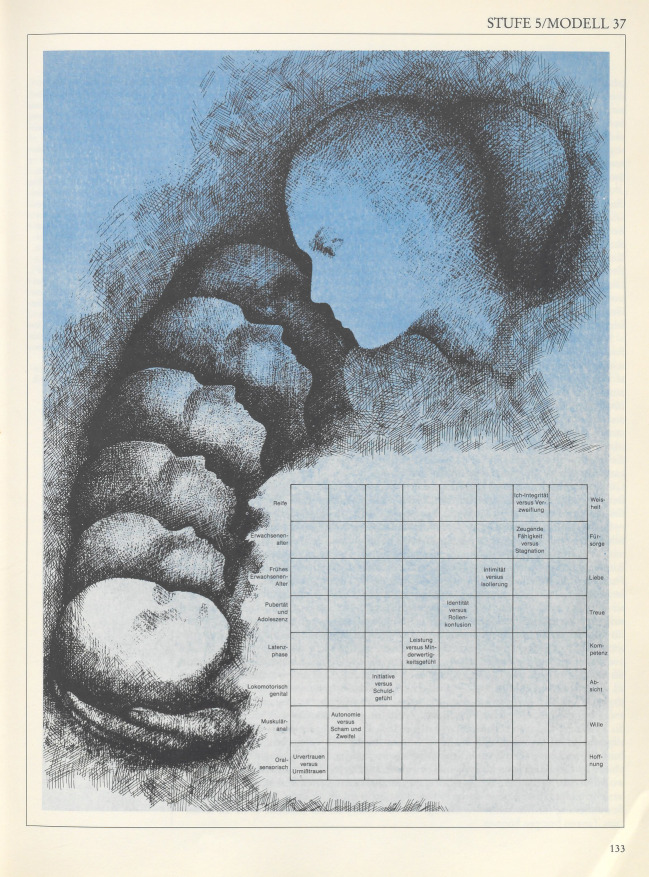

Als ein Leitfaden für Mütter beschrieben die „Acht Stufen“ die Kindererziehung als wesentliche Aufgabe der Frau (Abb. 2). Sie sollte in ihren Kindern, insbesondere den Jungen, Vertrauen und Eigenständigkeit stiften und sie zu anständigen Mitgliedern der Gesellschaft erziehen.

Diese Botschaft trat besonders deutlich zutage in Eriksons Beitrag zu einer Reihe interdisziplinärer Treffen, die die Macy Foundation zur Vorbereitung der Midcentury White House Conference on Children and Youth (1950) ausrichtete und die der Entwicklung von Erziehungsleitlinien für Eltern, Erzieher und Entscheidungsträger gewidmet waren. Erikson, der sein Phasenmodell hier erstmals in größerem Rahmen präsentierte, war der erste von vier eingeladenen Rednern, neben dem Psychoanalytiker Lawrence Frank, der Sozialpsychologin Marie Jahoda und dem Anthropologen Ashley Montagu; auch Ives Hendrick war unter den Diskussionsteilnehmern. Helen Witmer, die Leiterin des Untersuchungsausschusses, fand Eriksons Konzept des Lebenszyklus so sachdienlich, dass sie es ohne Änderungen in den Konferenzreport übernehmen wollte (Senn 1950: 38–39; zur Bedeutung der White House Conference für Eriksons Werk, siehe Friedman 1999: 229, 233).

Während des Treffens drehte sich die Diskussion über die „Acht Stufen“ weniger um Fragen der Kindesentwicklung als um die Aufklärung von Frauen und Müttern. Obwohl häufig von „Eltern“ oder „Müttern und Vätern“ die Rede war, war die Frage nach der Rolle des Mannes letztlich nachrangig. Wo sie thematisiert wurde, ging es um ein „Zuviel“ an Vaterschaft als Pathologie und Indiz für Homosexualität (siehe etwa Senn 1950: 62, 69–70). Im Gegensatz dazu beklagten die Diskussionsteilnehmer die mangelnde Identifikation von Frauen mit der Mutterrolle, imaginiert als Vollzeitbeschäftigung. Die Entwicklungspsychologin Lois Murphy, Gründerin der einflussreichen Sarah Lawrence Nursery School, die Kurse in frühkindlicher Erziehung anbot, fand Eriksons Ausführungen hilfreich, um ihren Studentinnen eine „innere Akzeptanz“ der Mutterrolle einzuflößen und ihnen auf diese Weise zu helfen, „ihre universelle [biologische] Bestimmung zu erfüllen“ (Senn 1950: 72–73).

Auch der bekannte Kinderarzt und Erziehungsratgeber Benjamin Spock, Autor des weit verbreiteten Ratgebers *Säuglings- und Kinderpflege *(1952, auch: *Dein Kind – dein Glück*) befürwortete die Betonung von Werten wie Empathie und Familienbeziehung in der Erziehung von Mädchen, sodass „die Vorstellung, einmal selbst Mutter zu sein, bereits während der Kindheit als ein spannendes Ziel erscheint.“ Für die Erziehung und Bildung im Jugend- und frühen Erwachsenenalter sprach sich Spock gegen Koedukation an Schulen und Universitäten aus, die er als schädlich für die weibliche Identifikation mit Häuslichkeit und Mutterschaft ansah: „Bildung für Frauen ist in weiten Teilen noch immer eine Imitation traditionell männlicher Bildung; sie lehrt Frauen, vor allem am College, Leistungen im Bereich der Geistes‑, Naturwissenschaften und Technik zu bewundern und zu ersehnen. Der Wert zwischenmenschlicher Beziehungen und der Kindererziehung wird demgegenüber geschmälert“ (Senn 1950: 74, 87; vgl. Weiss 1977; Apple 2006: 107–134).

Abweichung von der mütterlichen Rolle wurde als Fehlentwicklung pathologisiert. Zum Ende des Symposiums, im Anschluss an Marie Jahodas Präsentation („Zur Sozialpsychologie der psychischen Gesundheit“), beendete Erikson die Diskussion, indem er „Mütter [*sic*], die es ablehnen, Kinder zu bekommen“ als Inbegriff einer gescheiterten Identität porträtierte:

Es liegt hier ein gängiges Problem vor, nämlich das des amerikanischen Mädchens, das selbstbeherrscht, gesund und zupackend ist, solange es sich nicht mit seiner Mutter in der Mutterrolle identifizieren muss und dessen Identität auf, sagen wir, einem schlanken Körper, körperlicher Aktivität usw. basiert. Sie wird der Meinung sein, dass Kinder zu bekommen die Grundlagen ihres Selbstverständnisses in Frage stellt. (Senn 1950: 293–294)

Wie Erikson seinem Publikum versicherte, sei dies jedoch eine gewöhnlich Rollenkrise und im ärztlichen und therapeutischen Gespräch in der Regel leicht zu kurieren: „[D]ie Beschwichtigung einer Hebamme und die Umbildung gesellschaftlicher Werte können einer jungen Frau helfen, sich zu verändern“ (Senn 1950: 294; vgl. May 1996: 127–149).

Doch viele Frauen wollten sich nicht in diesem Sinne „verändern“. Sie lehnten die von den Experten beschriebene traditionelle Mutterrolle ab und forderten die Möglichkeit, sich selbst zu verwirklichen – so wie Erikson es in seinem Stufenmodell dargelegt hatte. War bereits im Falle Winnicotts der Übergang von der Idealisierung der traditionellen Frauenrolle zu antifeministischer Kritik fließend, so illustriert Eriksons Verhalten gegenüber der Frauenbewegung den zutiefst reaktionären Charakter des analytischen Konzepts femininen Umwelt-Seins.

## Der innere Raum

In ihrem Bestseller *The Feminine Mystique *(dt. *Der Weiblichkeitswahn*) reklamierte die feministische Aktivistin und Publizistin Betty Friedan, die bei Erikson in Berkeley studiert hatte, sein Entwicklungsmodell für Frauen, einschließlich seines Begriffs der „Identitätskrise“. Das von Erikson geschilderte „Problem“ des „amerikanischen Mädchens“ habe im Kern nichts mit biologischer Veranlagung zu tun,

sondern mit der Frage nach Identität […]. Warum jedoch haben die Wissenschaftler bei den Frauen nicht dieselbe Identitätskrise festgestellt [wie bei Männern]? Glaubt man den alten Überzeugungen und dem neuen Weiblichkeitswahn, so lag den Frauen weder daran, herauszufinden, wer sie sind, noch daran, nach einer Identität zu streben. Der Frauen Schicksal sei ihre Anatomie, behaupteten die Weiblichkeitsverfechter, ihre Identität sei biologisch festgelegt. Aber ist sie das wirklich? (Friedan 1986 [1963]: 55, 57)

Indem Friedan darauf hinwies, dass Frauen ebenfalls eine „Identitätskrise“ durchmachten, hinterfragte sie das vielzitierte Freud’sche Axiom von der Anatomie als weiblichem Schicksal. Dabei bezog sie sich nicht nur auf die Psychologie des Individuums, sondern verwob diese mit einem breiteren, gesellschaftlichen Begriff der Krise, wenn sie schrieb:

Immer mehr Frauen [sind sich] einer Identitätskrise in ihrem eigenen Leben bewusst, einer Krise, die vor vielen Generationen einsetzte […] und nicht enden wird, ehe sie oder ihre Töchter einen bisher unbekannten Weg beschreiten und aus sich selbst und ihrem Leben das neue Leitbild machen, das so viele Frauen heute so dringend brauchen. (Friedan 1986 [1963]: 57)

Friedan fuhr fort: „Ich glaube, dass diese Krise einen Wendepunkt in der Entwicklung von einer ‚weiblich‘ genannten Unreife zu voller menschlicher Identität bedeutet. Ich glaube, dass Frauen diese Identitätskrise durchmachen mussten […], damit sie ganz und gar menschlich werden“ (Friedan 1986 [1963]: 57). Als Teil eines Reifungs- und Wachstumsprozesses verstieß die Emanzipation der Frau – auf individueller ebenso wie gesellschaftlicher Ebene – also nicht, wie Erikson und andere nahelegten, gegen die natürliche Ordnung, sondern instanziierte vielmehr die Prinzipien der menschlichen Entwicklung.

Erikson widersprach. Für ihn war die Frauenbewegung nichts als eine „gigantische Posse“ (Erikson 1963: 583). Auf einer großangelegten Konferenz über „Die Frau in Amerika“ (The Woman in America), die die American Academy of Arts and Sciences im Herbst 1963 im Licht der Publikation und des Erfolgs von Friedans Buch organisierte, hielt der Psychologe einen Vortrag über „Inner and Outer Space“ – eine Anspielung auf den „Wettlauf ins All“ und eine Verteidigung traditioneller Geschlechtertrennung. Erikson widersprach mehreren der anderen Rednerinnen und Redner – der Soziologin Alice Rossi, der Sozialpsychologin Lotte Bailyn und dem Historiker Carl Degler –, die Friedans Analyse ausdrücklich bestätigten (Rossi 1964: 613n; Degler 1964: 668–669; Bailyn 1964: 709n2). Rossis berühmter „unbescheidener Antrag“ („An Immodest Proposal“) auf „Gleichheit zwischen den Geschlechtern“ war nicht weniger als ein Aufruf dazu, den „feministischen Funken […] unter amerikanischen Frauen“ erneut anzufachen (Rossi 1964: 608).

Erikson hingegen, aus Friedans *Feminine Mystique* zitierend, vertrat die Auffassung, dass „die im Augenblick modische Diskussion darüber, ob und wie die Frau ‚völlig menschlich‘ werden könnte, wirklich eine kosmische Parodie“ sei (Erikson 1980 [1964]: 275).^14^ In seinem Beitrag über „Die Weiblichkeit und den inneren Raum“ stellte der Psychologe klar, dass sein Modell der Entwicklung für Männer und Männer allein galt. Die weibliche Persönlichkeitsbildung folge anderen Gesetzen:

[D]ie Identitätsbildung der Frauen [unterscheidet sich] kraft der Tatsache, dass ihr somatischer Grundplan einen ‚inneren Raum‘ beherbergt, dazu bestimmt, die Nachkommen erwählter Männer zu tragen, und damit eine biologische, psychologische und ethische Verpflichtung, für die Kindheit des Menschengeschlechts Sorge zu tragen. (Erikson 1980 [1964]: 279).

Auch Erikson verwendete das Konzept des Haltens in metaphorischer Weise, freilich ohne auf Winnicotts Ausführungen einzugehen. Er hob die weibliche Fähigkeit und Verpflichtung hervor, „auf unterschiedlichen Daseinsebenen […] die Dinge anzunehmen, zu besitzen, auch: zu warten, und ihre Emotionen zu kontrollieren“ („to accept, to ‚have and hold‘—but also to hold on and to hold in“) (1964: 600).

Der Behältnis-Charakter der Frau hatte Implikationen vor allem für Diagnose und Therapie. Die spezifischen Probleme der weiblichen Psyche und Identität resultierten für Erikson – der damit die ältere Definition weiblicher „Hysterie“ wiederbelebte (Rousseau 1993) – aus der „Leere“ ihres inneren Raums. Erikson legte eine „post-Freudianische“ Begründung der Geschlechterunterschiede vor, die er dezidiert vom Konzept des Penisneids unterschied (Erikson 1964: 587–588, 593–597). So sei weibliches Leiden nicht auf den Mangel eines männlichen Merkmals zurückzuführen, sondern müsse aus der spezifischen Anlage und Konstitution der Frau als Gefäß verstanden werden: „Der ‚innere Raum‘ ist der Ursprung weiblicher Verzweiflung […] Leere ist die Verdammnis der Frau […], wie sie jede Frau erfährt. [Von einem Mann] verlassen zu werden, bedeutet für sie, leer gelassen zu werden“ (Erikson 1964: 596).

Der Analytiker rechnete jedoch nicht nur mit Freud ab, sondern er wandte sich vor allem gegen Friedan und die Frauenbewegung. Beim zeitgenössischen Problem der amerikanischen Frauen handle es sich nicht um eine kollektive Identitäts- und Selbstfindungskrise, die aus den Begrenzungen der traditionellen Frauenrolle resultierte. Als Ausdruck innerer „Leere“ verweise die Frauenbewegung vielmehr auf eine unzureichende Identifikation mit der weiblichen Rolle als Ehefrau und Mutter – denn nur in dieser Rolle könnten Frauen, im wörtlichen Sinne, „Erfüllung“ finden. Und so bekräftigte Erikson letztlich ausdrücklich das Freud’sche Axiom, das Friedan in Frage gestellt hatte: „Behaupte ich also, dass die Anatomie das Schicksal ist? Ja, sie ist Schicksal […]. Die Grundmodalitäten der Hingabe und Beteiligung der Frau spiegeln natürlicherweise auch den Grundplan ihres Körpers wider“ (Erikson 1980 [1964]: 298–299).

Psychologiehistorikerinnen haben Eriksons Glorifizierung der Innerlichkeit als eine „entschieden pro-feministische“ Bekräftigung weiblicher Qualitäten missverstanden (Friedman 1999: 243); vermutlich, weil der Neo-Freudianer sich explizit von der Theorie des Penisneids distanzierte. So verteidigt Ellen Herman in ihrem Standardwerk *The Romance of American Psychology *den Analytiker als Vorläufer feministischer *Care*-Ethik; der Erikson-Biograph Friedman legt eine ähnliche Interpretation vor (Herman 1996: 293, vgl. ebd.: 391n78; Friedman 1999: 243–246).^15^ Dabei war Eriksons Modell der besonderen weiblichen Entwicklung eine Erwiderung auf Friedan und, letztlich, eine Reaktion gegen die Frauenbewegung überhaupt.

Friedan antwortete nicht noch einmal auf Erikson, doch andere schalteten sich zahlreich ein: feministische Expertinnen und Publizistinnen, vom *radical caucus* der American Psychiatric Association (APA) über die Psychologin Naomi Weisstein bis zur australischen Autorin und Aktivistin Germaine Greer. Kate Millett widmete Erikson ein ganzes Unterkapitel in ihrem Erfolgsbuch *Sexual Politics *(1970) und problematisierte den „galanten Chauvinismus“ des Analytikers Jahrzehnte bevor er Historikerinnen in die Irre leiten sollte. Sie wies Eriksons Text als eine aktualisierte Version von Freuds biologischem Determinismus aus und hielt fest, dass der

beunruhigende und bisweilen widersprüchliche Ton des Aufsatzes zu einem Großteil daher rührt, dass Erikson zwischen zwei Frauenbildern schwankt, nämlich Freuds Chauvinismus und seiner eigenen Art der Ritterlichkeit. Er möchte daran festhalten, dass die weibliche Anatomie Schicksal (und auch Persönlichkeit) sei. Gleichzeitig jedoch plädiert er dafür, die vorbestimmte, historische Unterordnung der Frau durch ein galantes Zugeständnis an die Ansprüche der Mütter zu mindern sei. (Millett 1970: 212; vgl. Schmidt 2020: 61–63)

Noch Ende der 1970er Jahren erinnerte – und evozierte – die Literaturwissenschaftlerin Cynthia Griffin Wolff „das Entsetzen, das Frauen ergriff (und dem sie Ausdruck verliehen), die Eriksons Essay lasen und feststellen mussten […], dass seine schönen Worte letztlich zu jenen Gemeinplätzen über ‚Weiblichkeit‘ führten, gegen die sie so lange gekämpft hatten“ (Wolff 1979: 356).

Der inhärente Antifeminismus Eriksons und Winnicotts speiste sich nicht zuletzt aus dem Konzept femininen Umwelt-Seins. Dies implizierte zum einen die Kopplung männlicher Individuation an traditionelle Weiblichkeit – Winnicott sprach von „Abhängigkeit“ (Winnicott 1969j [1957]; ders. 1974). Eine Veränderung in der Rolle der Frau drohte in dieser Logik das Wohlbefinden des Mannes zu beeinträchtigen und gefährdete gar das Gemeinwohl. Zum anderen ging mit der Annahme weiblicher Konstanz eine Pathologisierung des Wandels der Rolle der Frau einher. Eriksons Modell der Persönlichkeitsentwicklung erklärte nicht einfach den männlichen Lebenslauf zum allgemeinen Maßstab, demgegenüber Frauen „zurückblieben“, wie Psychologinnen wiederholt kritisiert haben (Gilligan 1982; Sorrell & Montgomery 2001: 113–119; Worrell & Remer 2002: 90). Wie seine Reaktion gegen Friedans Übertragung seiner Stufentheorie auf den weiblichen Lebenslauf zeigt, schloss er Frauen aus seinem Modell der Persönlichkeitsentwicklung kategorisch aus. Die Ideale des Wachstums und der Autonomie, der Leistung und Integrität waren Männern vorbehalten. Das Konzept der Environmentalität nahm Frauen von der Entwicklung aus und verwehrte ihnen auf diese Weise die Möglichkeit, ihr Leben zu verändern. Diese Eigenschaften machten das analytische Umweltdenken zu einem nützlichen Instrument für die antifeministische Gegenreaktion, die um 1980 weithin Anklang fand.

## Krise der Männlichkeit

Seit den 1970er Jahren wandten Psychologen den Entwicklungsbegriff zunehmend auf das Erwachsenenalter an und lieferten auf diese Weise wirkmächtige Erklärungen für den sozialen und gesellschaftlichen Wandel, der das Jahrzehnt kennzeichnete. Zugleich legten sie mit der Weiterentwicklung des post-freudianischen Denkens – häufig verbanden sie verschiedene Strömungen miteinander – und insbesondere unter Rückgriff auf Konzepte weiblichen Umwelt-Seins eine Antwort auf die feministische Kritik an der Psychoanalyse vor, die in den 1960er und 1970er Jahren zunehmend Gehör fand (Buhle 1998; Herzog 2017: 68–72). In seiner vielzitierten Studie zum *Leben des Mannes* (1979) verwendete der Sozialpsychologe Daniel Levinson (1920–1994) – ein weiterer Erikson-Schüler und Kommilitone Friedans (Friedan 1993: 110) – die „Acht Stufen“ als Ausgangspunkt seines Lebenslaufmodells und übertrug Winnicotts Modell der Mutter-Kind-Beziehung auf Ehe und Partnerschaft.

Zwar nahm Levinson auf den Begriff der „fördernden Umwelt“ nur selten explizit Bezug, auch wenn er dafür plädierte, Winnicotts Begriff auf die Erwachsenenentwicklung anzuwenden (Levinson 1978: 346n36). Doch seine Kritik an feministischen Positionen war getragen vom Verständnis weiblicher Environmentalität – insbesondere in Bezug auf Ehe und Partnerschaft – und seiner Bedeutung für das Wohlbefinden eines Mannes. *Das Leben des Mannes *war Teil eines breiten gesellschaftlichen „Backlashs“ gegen die Frauenbewegung um 1980, der sich häufig psychologischer Argumente bediente (Faludi 2006: 345–372). Deutlicher noch als Winnicott und Erikson buchstabierte Levinson die Befürchtung aus, die traditionelle, binäre Theorien der Identitätsbildung verfolgte. Wenn Frauen den ihnen zugewiesenen Platz verließen, würde – in den Worten der Kritikerin Susan Faludi – das „zarte Pflänzchen der Maskulinität“ verkümmern: „Nichts scheint die Blütenblätter der Männlichkeit so sehr zu zerdrücken wie ein wenig feministischer Regen – schon wenige Tropfen werden als Wolkenbruch wahrgenommen“ (Faludi 2006: 76).

*Das Leben des Mannes*, ursprünglich unter dem Titel *The Seasons of a Man’s Life *(1978) beim Publikumsverlag Alfred Knopf erschienen und binnen eines Jahres auf Deutsch von Kiepenheuer & Witsch publiziert, wurde nicht nur von einer breiten Leserschaft begeistert aufgenommen, sondern bleibt bis heute eine der grundlegenden fachwissenschaftlichen Publikationen zum Thema der männlichen Midlife-Crisis. Das Buch basierte auf einem vom National Institute of Mental Health (NIMH) geförderten Forschungsprojekt zur „Psychosoziologie der männlichen Lebensmitte“ (A Psychosocial Study of the Male Mid-Life Decade, 1968–1973), das leitende Interessen aus Levinsons bisheriger Arbeit vereinte: zum einen die Konformismuskritik, mit der der Psychologe durch die Mitarbeit an *Die autoritäre Persönlichkeit* (1950) während seiner Zeit als Doktorand im Rahmen der Berkeley Public Opinion Study Group (1944–1947) vertraut war und die nicht nur Levinsons Arbeit, sondern die zeitgenössischen amerikanischen Sozialwissenschaften überhaupt prägte (Adorno et al. 1950; Cohen-Cole 2014: insbesondere 35–62).^16^ Zum anderen verschob sich Levinsons Forschungsinteresse in den 1950er und 1960er Jahren auf das Feld der Organisations- und Managementpsychologie, dem er sich zunächst in Harvard und ab 1966 an der Yale University widmete. Sein Hauptinteresse galt der mittleren und höheren Führungsebene und insbesondere Fragen der Karriereentwicklung (Hodgson et al. 1965). Jene „Männer in grauen Flanellanzügen“, die Soziologen wie David Riesman, C. Wright Mills oder William Whyte als Inbegriff der Angepasstheit erschienen, wurden in Levinsons Darstellung zu rebellischen Helden und Playboys.^17^

Die „Male Mid-Life Study“ beschrieb die Lebensmitte als eine Periode der Veränderung, in der Männer ihr Leben überdachten und sich neu erfanden. Levinson und sein Team – die Psychologin Maria Hertz Levinson (Daniels damalige Ehefrau) und der Psychologe Edward Klein, der Psychiater Braxton McKee und die Soziologin Charlotte Darrow – erhoben und analysierten die Lebensgeschichten von vierzig Männern mittleren Alters. Jeweils zehn von ihnen waren leitende Angestellte bei den in Connecticut ansässigen Chemie- und Elektro-Unternehmen *Olin* und *GSI*, Arbeiter (bei denselben Unternehmen), Biologen und Schriftsteller. Fünf der Teilnehmer waren schwarz: drei Arbeiter und zwei Schriftsteller. Trotz des vergleichenden Ansatzes seiner Studie interessierte sich Levinson kaum für die Unterschiede im Leben der Männer. Es ging ihm vielmehr darum, zu zeigen, auf welche Weise das Leben der Arbeiter, Naturwissenschaftler und Schriftsteller den Lebensläufen der Manager ähnelten. Für Levinson verwirklichte der Karriereweg der Direktoren grundlegende Prinzipien der Entwicklungspsychologie im Allgemeinen und der männlichen Persönlichkeitsentwicklung im Besonderen. Die zentrale Fallstudie in *Das Leben des Mannes* war denn auch der Vizepräsident und Hauptgeschäftsführer der *Olin*-Schusswaffenabteilung, unter dem Pseudonym „Jim Tracy“. Nach zahlreichen Seitensprüngen und Affären – er las Frauen auf „wie [andere] Kiesel am Strand“ (1978: 118) – verließ Tracy seine Frau und das gemeinsame Kind und heiratete eine jüngere Frau, bevor er seinen Job bei *Olin* kündigte, um ein eigenes Unternehmen zu gründen und endlich ganz er selbst, „sein eigener Chef“ zu werden (ebd.).

Levinsons Schilderung schien mehr oder weniger aus dem *Playboy *kopiert, doch der Psychologe meinte es ernst: Tracys Verhalten, so Levinsons Hauptargument, zeuge von einem wichtigen Entwicklungsschritt, dem „Lebensmitte-Wechsel“ oder der „Midlife-Crisis“. Unter Berufung auf Erikson charakterisierte Levinson die Loslösung eines Mannes von seiner Frau und Familie als Zeichen von Reife, Erkenntnis und Integrität:

[In der Lebensmitte] kann ein Mann endlich erkennen, dass die Ehe von Anfang an Mängel hatte. Er hat nicht aus großer Liebe, sondern aus anderen Gründen geheiratet, sei es, dass die Familie ihn drängte oder dass die Konvention es erforderte, dass er aufbegehrte, sich den sozialen Aufstieg erhoffte oder einfach Schuldgefühle hatte. […] Nun ist plötzlich der große Nebel der Illusion verflogen. (Levinson 1979: 352)

Levinsons Konzept der Midlife-Crisis bezog sich ausschließlich auf Männer – Frauen wurden lediglich befragt, um mehr über das Leben ihrer Ehemänner zu erfahren (Levinson 1978: 13). Der Psychologe äußerte sich widersprüchlich über die Anwendbarkeit seines Entwicklungsmodells auf den weiblichen Lebenslauf. Zwar bezeichnete er *Das Leben des Mannes *als die unumgängliche „Grundlage einer Studie über Frauen“ (Levinson 1978: 9). Doch zugleich schloss Levinson Frauen von seiner Studie aus, mit der Begründung, dass die Unterschiede zwischen den Geschlechtern für eine parallele Analyse zu groß seien. Vergleiche zwischen Menopause und Midlife-Crisis wies er als unbrauchbar, gar lächerlich von der Hand (Levinson 1978: 24).^18^ Dennoch war die Midlife-Crisis untrennbar verbunden mit Vorstellungen über die Rolle der Frau. Ähnlich wie bereits bei Erikson nahmen Frauen gerade in ihrer Abwesenheit eine tragende Rolle in Levinsons androzentrischer Studie ein: als konstitutive Bedingung des guten „Lebens des Mannes“.

Der Psychologe übertrug Winnicotts Darstellung der Umwelt-Mutter auf die Ehefrau und deren Bedeutung für den beruflichen Erfolg und das Wohlbefinden des erwachsenen Mannes. Dabei war es Levinson – im Unterschied zu Winnicott wie auch Erikson – freilich weniger um Mutterschaft und Kindesentwicklung zu tun als um Ehe und Partnerschaft.^19^ Die Ehefrau stellte, noch vor den im Kollektivsingular auftretenden, austauschbaren Geliebten, die zentrale Frauenfigur im *Leben des Mannes *dar. Levinson unterstrich ihre Bedeutung für die Zufriedenheit und den beruflichen Erfolg eines Mannes. Seine Definition der Tugenden der idealen Ehefrau gemahnte an Winnicotts Beschreibung der „normalen“, „hingebenden“ Umwelt-Mutter. So identifiziere sich die „besondere Frau“ (*special woman*) vollständig mit den Zielen und Wünschen ihres Ehemannes. In „erster Line Ehefrau und Mutter“, sei sie „generell mütterlich und fürsorglich (*caring*) und erleichtert ihm das Leben“ (Levinson 1978: 248); ihr Selbstwert speise sich aus dem Wohlergehen ihres Mannes, den sie als einen „Held“ verehre, und ihr oberstes Ziel sei, seine Bedürfnisse zu erfüllen und ihm bei der Verwirklichung seiner Pläne behilflich zu sein. Und genauso wie Winnicotts Mutter war auch Levinsons „besondere Frau“ letztlich eine „Übergangsfigur“ im Leben eines Mannes. So zentral sie für seine Entwicklung im jüngeren Erwachsenenalter sei, so wachse er doch mit zunehmendem Alter über sie hinaus. Im mittleren Alter werde die „besondere Frau“ nicht nur überflüssig, sondern stehe der Entfaltung eines Mannes tatsächlich oft im Wege (Levinson 1978: 109). Tatsächlich sah er ihre „besonderen“ Eigenschaften nun als „allzu sehr kontrollierend“, „einengend“, gar „erniedrigend“ an (Levinson 1978: 256).

Der Psychologe verlieh einer antifeministischen Haltung mindestens ebenso klar Ausdruck wie seine Vorgänger. Auf dem Backlash-Panel der *Vogue *erklärte Levinson: „Frauen sollten nicht zu viel Autorität haben – weder zu Hause noch im Arbeitsleben; das macht alle nervös“ (Rayon 1986: 305). Deutlich wandte er sich gegen Versuche, die Rolle der Frau zu überdenken und neu zu definieren, wenn er, in *Das Leben des Mannes*, der besonderen, normalen und hingebungsvollen Hausfrau die „emanzipierte“ Frau gegenüberstellte. Ihre Unabhängigkeit und berufliche Einbindung stünden dem Lebensglück eines Mannes entschieden im Wege und seien tatsächlich wider die menschliche Natur und Kultur: „Es ist schwierig genug, eine Lebensstruktur auf dem ‚Traum‘ einer Person aufzubauen. Eine Struktur aufzubauen, die die ‚Träume‘ beider Partner enthalten kann, ist tatsächlich eine gewaltige Aufgabe, auf die Evolution und Geschichte uns nur unzureichend vorbereitet haben“ (Levinson 1978: 110).

Im Rückgriff auf einen klassischen Topos reaktionärer, antifeministischer Rhetorik deklarierte Levinson eine „Krise der Männlichkeit“, ausgelöst durch eine Veränderung der Rolle der Frau. Im Kontext von Studien zur Maskulinität ist vermehrt darauf hingewiesen worden, dass das Narrativ einer „Krise der Männlichkeit“ unweigerlich die Annahme einer stabilen, gewissermaßen essenzialisierten Männlichkeit zum Ausdruck bringt, markiert der Begriff der Krise doch „eben nicht den Ausnahme-, sondern den Normalzustand der Bedeutungsgebung“ (Kaltenecker 2000: 42). In diesem Sinne impliziere der Begriff einer Krise der Männlichkeit „ein kohärentes System, das zunächst einmal positiv besetzt und von einer ‚authentischen‘, wünschenswerten Form von Männlichkeit getragen ist und das dann durch diese Krise zer- bzw. gestört wird“ (Martschukat & Stieglitz 2008: 64; vgl. Opitz-Belakhal 2008; Connell 2015: 138–139).^20^

Übersehen wird dabei, dass die „Krise der Männlichkeit“ sich nicht allein auf Männer bezieht, sondern maßgeblich das Leben von Frauen betrifft. Tatsächlich war die Deklaration einer „Krise der Männlichkeit“ ein erprobtes Instrument im Kampf gegen feministische Forderungen nach einem Wandel der Geschlechterrollen (Faludi 2006: 77–78). In diesem Sinne war Levinson der Ansicht, dass die Emanzipation der Frau Männer daran hindere, all ihre Möglichkeiten auszuschöpfen. In *Das Leben des Mannes* schrieb er, dass „das Bestreben [der Frau], von einer vorwiegend häuslichen Rolle wegzukommen, […] ihren Horizont zu erweitern und sich nach Tätigkeiten außerhalb des Hauses umzusehen“ den Werdegang und die Selbstverwirklichung von Männern beeinträchtigen würde:

Manchmal ist es die Frau, die den ersten Schritt zu einer Neubeurteilung der Ehe unternimmt. […] Sie wird zur Stimme der Entwicklung und Veränderung. […] [Der Mann wird] nun zur Stimme des Status quo. [Er] fühlt sich […] bedroht. […] Wenn die Frau selbstbewusster und freier wird, kommt es […] beim Mann zu einer schweren Krise. (Levinson 1979: 355)

Der Topos von den Beschwerden des Mannes – „der sich als entmachtet wahrnimmt, weil es ihm nicht gelungen ist, Frauen gänzlich zu unterdrücken“ (Levy 2018: 113) – zementiert und erzeugt in diesem Sinne nicht nur die Hegemonie einer angeblich bedrohten Maskulinität. Vielmehr dient die „Krise der Männlichkeit“ insbesondere dazu, die Unveränderlichkeit der Rolle und Stellung der Frau einzufordern.

## Environmentalität und Antifeminismus

Psychologische Modelle der Persönlichkeitsentwicklung, das wird aus historischer Perspektive klar, sind gesellschaftliche und politische Entwürfe, die das Leben von Frauen auf besondere Weise betrafen und betreffen. Bis heute gilt die Entwicklung des Kindes gemeinhin als Inkarnation mütterlicher Kompetenz: Sie „reflektiert das Wissen der Mutter. […] Das Kind zieht die Mutter heran“ (Hays 1995: 45; Zitat: Strathern 2005: 5). Zwar privilegierten die analytischen und psychologischen Entwicklungsmodelle des 20. Jahrhunderts das Leben des Mannes. Dabei schlossen sie Frauen jedoch nicht einfach aus, sondern konzipierten sie vielmehr als das konstitutive „Außen“ primär männlich definierter Entwicklung und Zufriedenheit. Wenn Winnicott die ideale Frau und Mutter als „ermöglichende Umwelt“ bezeichnete und sie damit zu Fürsorge, Geduld, Beständigkeit und Treue anhielt, so kontrastierte die Statik der Umwelt-Mutter oder Umwelt-Frau mit Beschreibungen des männlichen Wachstums und der Entwicklung und demonstrierte zugleich die Relevanz von Gender und *care work *für Konzeptionen von Mensch und Umwelt.

Indem sie Normen der Entwicklung entwarfen, forderten Psychologen und Analytiker Frauen dazu auf, als Mütter und Ehefrauen Umwelten hervorzubringen und Bedingungen sicherzustellen, die das Wachstum ihrer Kinder und den Erfolg der Ehemänner ermöglichen sollten. Im Konzept weiblicher Environmentalität wurden Frauen gar selbst als Milieu begriffen und konstituiert: anwesend, unveränderlich und verfügbar. Winnicott formulierte diese Annahmen in seiner Beschreibung der „Umwelt-Mutter“ aus, Erikson setzte sie voraus und Levinson übertrug sie auf Ehe und Partnerschaft. Ob ausgesprochen oder angenommen, der Umweltbegriff war im Entwicklungsdenken fest verankert und eindeutig weiblich konnotiert. Gegenüber ökofeministischen Idealisierungen der Nähe von Frau und Natur illustriert der entwicklungspsychologische Diskurs damit eine konservative, latent reaktionäre Konzeption femininer Environmentalität, eingefordert als Bedingung männlichen Werdens und Seins.

Im familiären und emotionalen Kontext erscheint die „Umwelt“ als eine Vorschrift und ein Befehl, der Frauen dazu aufrief, ein Milieu für die Erfüllung männlicher Hoffnungen und Bestrebungen zu erzeugen, erhalten und gar zu verkörpern. Dieses Konstrukt der Persönlichkeitsentwicklung fixierte Frauen in Zeit und Raum – oft gegen deren Willen oder auf Kosten ihrer Eigenständigkeit und ihres Wohlergehens. Der defensive und reaktionäre Gebrauch der Theorien in Auseinandersetzungen über die Emanzipation der Frau demonstriert die fest verankerte antifeministische Annahme, die diesen leitenden Konzeptionen der Identität und Selbstverwirklichung eingeschrieben ist: Das Leben und die Vorstellungen von Frauen hatten sich nicht zu verändern. Die Frau sollte Entwicklung und Individuation ermöglichen, war – als „Umwelt“ – selbst jedoch zum Stillstand verdammt.

## Footnotes

1 Zum atmosphärischen, affektiven Umweltbegriff und zu feminisierter Reproduktionsarbeit, vgl. Käthe von Boses Ausführungen in diesem *Special Issue* sowie auch Hochschild (1983).

2 Hier und im Folgenden ist das Jahr der Erstpublikation der Winnicott-Gesamtausgabe entnommen (Winnicott 2017a). Im gesamten Text wird, soweit möglich, aus den deutschen Übersetzungen zitiert; im Übrigen handelt es sich um Übersetzungen der Autorin.

3 Auch andere Psychoanalytiker wie John Bowlby und Anna Freud traten in der BBC auf, vgl. Shapira (2013: 115n13).

4 Vgl. demgegenüber die Verwendung der Gartenmetapher als Ausdruck weiblicher Kreativität im Kontext der Diskriminierung schwarzer Frauen in den USA bei Alice Walker (1983).

5 Ähnliche Momente weiblicher Umwelt-Verkörperung beobachtet auch Lisa Malich in ihrem Beitrag in diesem *Special Issue* in psychoanalytischen und medizinischen Konzeptionen von Schwangerschaft seit dem Zweiten Weltkrieg.

6 In ähnlicher Weise wird auch Erik Erikson als „optimistisch“ apostrophiert (Illouz 2008: 158–159).

7 In den 1980er Jahren wurde Superwoman zu einem Topos antifeministischer Publizistik, die den Versuch, Mutterschaft und Beruf zu vereinbaren, kritisierte, vgl. Faludi (2006: 57–60); Schmidt (2020: 151–152, 156–157). Zum „Superwoman-Schema“ und zum Klischee der „strong black woman“ als Ausdruck der Diskriminierung schwarzer Frauen in den USA, siehe auch Woods-Giscombé (2010).

8 Winnicott beschrieb das *transitional object* – klassischerweise ein Deckenzipfel oder Kuscheltier – freilich als Mutter-Substitut. Dennoch stand es im Zeichen der Distanzierung von der Mutter (Winnicott 1969i [1951]; Dever 1998: 69; Jacobs 1995: 53–54, 106). Eine ähnliche Konzeption der Frau als Medium der Männlichkeit findet sich auch in der Medien- und Techniktheorie Paul Virilios: „Der Mann ist der Passagier der Frau, nicht nur bei der Geburt, sondern auch in sexuellen Beziehungen […]. [Man könnte] sagen, dass das Weibchen das Mittel ist, welches das Männchen gefunden hat, um sich zu reproduzieren, das heißt, um auf die Welt zu *kommen*. In diesem Sinne ist die Frau das erste Transportmittel der Gattung [Mensch], ihr erstes Fahrzeug“ (Virilio 1978: 74).

9 Der Text „Further Thoughts on Babies as Persons“ wurde nicht in die deutsche Übersetzung von *The Child, the Family and the outside World *(1964a) aufgenommen. Er erschien ursprünglich 1947 unter dem Titel „Babies are Persons“ in *New Era in Home and School *(Winnicott 1947).

10 Siehe dazu insbesondere die Literatur zu Schwangerschaft als Krise: Duden (1991); Kukla (2005); Lyerly et al. (2009); Kukla (2010); Lupton (2012); Menkedick (2020: 25–58).

11 Auch Tellenbach spricht über die „Atmosphäre ausstrahlende“ Mutter, die das „Wachsen [des Kindes] umhüllt“ (1968: 51), und bezieht sich dabei vor allem auf Erik Erikson und René Spitz (Tellenbach 1968: 49–53).

12 Winnicott verwies hier auch auf den Beitrag seiner Ehefrau, der Sozialarbeiterin und Psychoanalytikerin Clare Britton Winnicott, zum Konzept des Haltens in der Beratungspraxis (Winnicott 2004 [1955]).

13 Winnicott sprach während der 1960er Jahre regelmäßig vor der Progressive League; auf den Feminismus-Vortrag folgte anlässlich der Konferenz „Men, Women, and the Future“ (1969) eine Rede über die Anti-Baby-Pille (2009b [1969]; vgl. Winnicott’s Lectures 2017; Coles 1971).

14 Vgl. die frühere Version des Textes: Erikson (1964).

15 Siehe demgegenüber die Erikson-Kritik einer der prominentesten *Care*-Feministinnen, der Psychologin Carol Gilligan (1982: 11–15).

16 In *The Authoritarian Personality *zeichnete Levinson verantwortlich für die Skalen zur Messung von Antisemitismus, Ethnozentrismus und politischem und ökonomischem Konservatismus (A–S-, E‑ und PEC-Skalen), die psychologische Ideologie-Analyse und die zusammenfassende Auswertung der Projektionsfragen sowie für Fragen des statistischen Vorgehens und Designs (Adorno et al. 1950: xiii, 57–207, 280–288, 545–600).

17 Wilson (1955); Riesman (1950); Mills (1951); Whyte (1956). Zu Nonkonformität und Unternehmenskultur vgl. Frank (1997); zu Angestelltenkultur und Playboy-Eskapismus: Martschukat (2011).

18 In seiner posthum veröffentlichten Studie zum „Leben der Frau“ argumentierte Levinson, dass es für Frauen unmöglich sei, den Sinn ihres Lebens in Arbeit und Beruf zu finden (Levinson 1996).

19 Kira Jürjens verweist in ihrem Beitrag in diesem *Special Issu**e* auf einen ähnlich fließenden Übergang zwischen der „bildenden“ Rolle der Mutter und der Ehefrau, die in Einrichtungstexten des 19. Jahrhunderts noch über den Familienkreis hinaus auch auf Kultur und Nation bezogen wurde.

20 Siehe demgegenüber den diagnostischen Begriff einer „Krise der Männlichkeit“, etwa bei Badinter (1993).
